# Detection of genetic variation using dual-labeled peptide nucleic acid (PNA) probe-based melting point analysis

**DOI:** 10.1186/s12575-015-0027-5

**Published:** 2015-11-04

**Authors:** Deokhwe Hur, Myoung Sug Kim, Minsik Song, Jinwook Jung, Heekyung Park

**Affiliations:** SeaSun Biomaterials, N517 Deadoek Campus, Pai Chai University, 11-3 Tekeuno 1-ro, Gwanpyeong-dong, Yuseong-gu, Deajeon 305-509 South Korea; Pathology Division, National Fisheries Research and Development Institute, Busan, 619-902 South Korea

**Keywords:** Peptide Nucleic Acid (PNA), Fluorescence probe-based melting point analysis, Real-time PCR, Genotyping, Mutation, Probe hybridization, Multiplex

## Abstract

**Background:**

Thermal denaturation of probe-target hybrid is highly reproducible, and which makes probe melting point analysis reliable in the detection of mutations, polymorphisms and epigenetic differences in DNA. To improve resolution of these detections, we used dual-labeled (quencher and fluorescence), full base of peptide nucleic acid (PNA) probe for fluorescence probe based melting point analysis. Because of their uncharged nature and peptide bond-linked backbone, PNA probes have more favorable hybridization properties, which make a large difference in the melting temperature between specific hybridization and partial hybridization.

**Results:**

Here, we have shown that full base dual-labeled PNA is apt material for fluorescence probe-based melting point analysis with large difference in the melting temperature between full specific hybridization and that of partial hybridization, including insertion and deletion. In case of narrowly distributed mutations, PNA probe effectively detects three mutations in a single reaction tube with three probes. Moreover, we successfully diagnose virus analogues with amplification and melting temperature signal. Lastly, Melting temperature of PNA oligomer can be easily adjusted just by adding gamma-modified PNA probe.

**Conclusions:**

The PNA probes offer advantage of improved flexibility in probe design, which could be used in various applications in mutation detection among a wide range of spectrums.

**Electronic supplementary material:**

The online version of this article (doi:10.1186/s12575-015-0027-5) contains supplementary material, which is available to authorized users.

## Background

The introduction of advanced DNA sequencing technology has made a remarkable improvement in discovering the genetic variations or mutations in the human genome, virus, bacteria, and plants [1–3]. Detection of Single Nucleotide Polymorphisms (SNPs) and short insertions and deletions (indels) is a common goal in high-throughput sequencing experiments. Indel variations, the most common type of structural variance in the genome of an organism, affect a multitude of traits and diseases in the organism. Moreover, identification of genotypes in organisms using species- and genotype-specific DNA markers is very useful for species identification, diagnostics, and breeding and preservation programs. New sequencing technologies, such as deep sequencing, allow massive throughput of sequence data and greatly contribute to genetic variation studies.

Despite the advances of new sequencing technologies, we still face a limitation in the number of screening technologies, which include probe-based real-time PCR, specifically chip or sequencing [4–6]. Real-time PCR assays using fluorescence resonance energy transfer (FRET) probes, molecular beacons, or TaqMan probes have been adapted for continuous mutation detection of amplification products in a closed system. Nevertheless, these assays do not effectively distinguish the differences between wild type and mutant types of SNP(s), insertion(s), or deletion(s) because of several handicaps [7]. First of all, traditional probe systems that have been used in SNP detection use two or more fluorescence channels for one mutation loci. When it comes to more than quadruplex detection, it can be a major problem in using real-time PCR. Moreover, traditional methods require ether the use of modification bases, special enzymes, or additional proprietary reagents or procedures. To solve these problems, we have adopted dual-labeled (fluorescence and quencher), full base peptide nucleic acid (PNA) hybridization probes for characterization of mutation assays [8, 9].

PNAs are artificially synthesized DNA analogs with an uncharged peptide backbone [10]. They have more favorable hybridization properties and chemical, thermal, and biological stability because of their uncharged nature and their peptide bond-linked backbone [9]. Because of these favorable characteristics, PNA probes are designed shorter (9–13 bp) than DNA probes with the same melting temperature (Tm). These characteristics make PNA probe more acceptable to use in mutation detection studies because PNA probe makes a large difference in ΔTm between perfect match and single mismatch, including even insertion and deletion. In this study, dual-labeled PNA probes were used to analyze genetic mutations including SNP, insertion, and deletion.

## Results

### Description of dual-labeled PNA probe-based FMCA for SNP genotyping

**Fig. 1:**
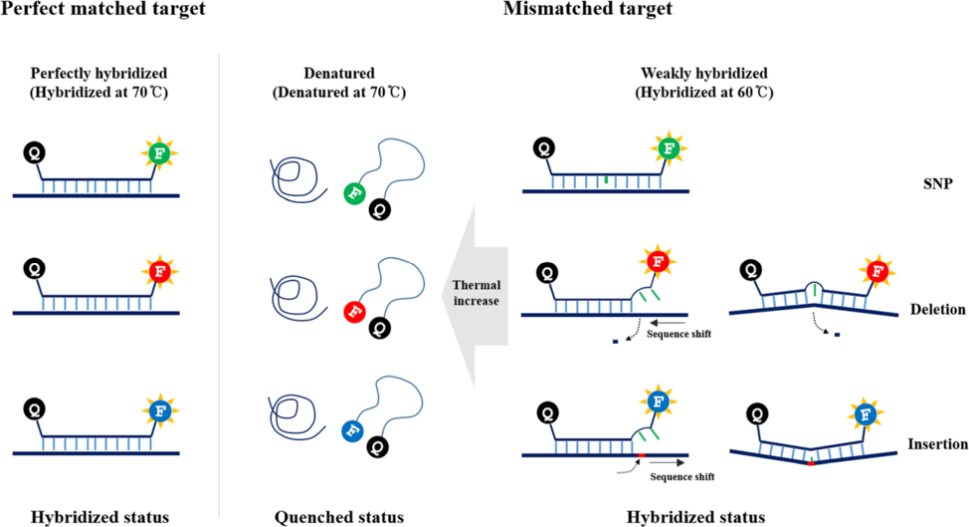
PNA probe-based FMCA for mutation detection. Schematic model of PNA probe-based FMCA for detection of SNP, deletion, and insertion. Mismatch or partial hybridization makes probe melting temperature lower than that of perfectly matched probe. At the denatured temperature, the PNA probe undergoes fluorescence quenching by random coiling and this quenching temperature was analyzed by the real-time PCR machine. Blue line, perfectly matched sequence; Green line, mismatched or unmatched sequence; Red line, inserted sequence; Q, quencher; F, Fluorescence

Generation of fluorescence signals during hybridization, performed using the dual-labeled full base PNA probe-based melting point analysis, is illustrated in Fig. 1. PNA probe is a typical, dual-labeled, random coiled quenching probe. It is a linear oligonucleotide consisting of a fluorescence covalently attached to the 3’-end and a quencher at the 5’-end. The randomly coiled structure enables fluorescence quenching until the probe is hybridized [9]. Therefore, non-hybridized PNA probe is only weakly fluorescent but becomes strongly fluorescent when hybridized with its target. We hybridized a PNA probe to partially specific oligonucleotide target and examined the hybrids for thermal denaturation. The results showed that fluorescence intensity of the hybrids decreased as the temperature increased in a target-dependent manner (Additional file 1: Figure S2, left panel), yielding different melting point (Tm) values for each target derived from the melting point (Additional file 1: Figure S2, right panel). The PNA probe designed for detection of three types of variation (SNP, deletion, and insertion) with sequence shift (causes mismatches at the end of the probe) and structural changes that result from small insertion or deletion at the center of the probe, which renders probe-binding efficiency lesser than that of perfectly matched probe (Fig. 1 and Additional file 1: Figure S2).

### SNP detection

**Fig. 2:**
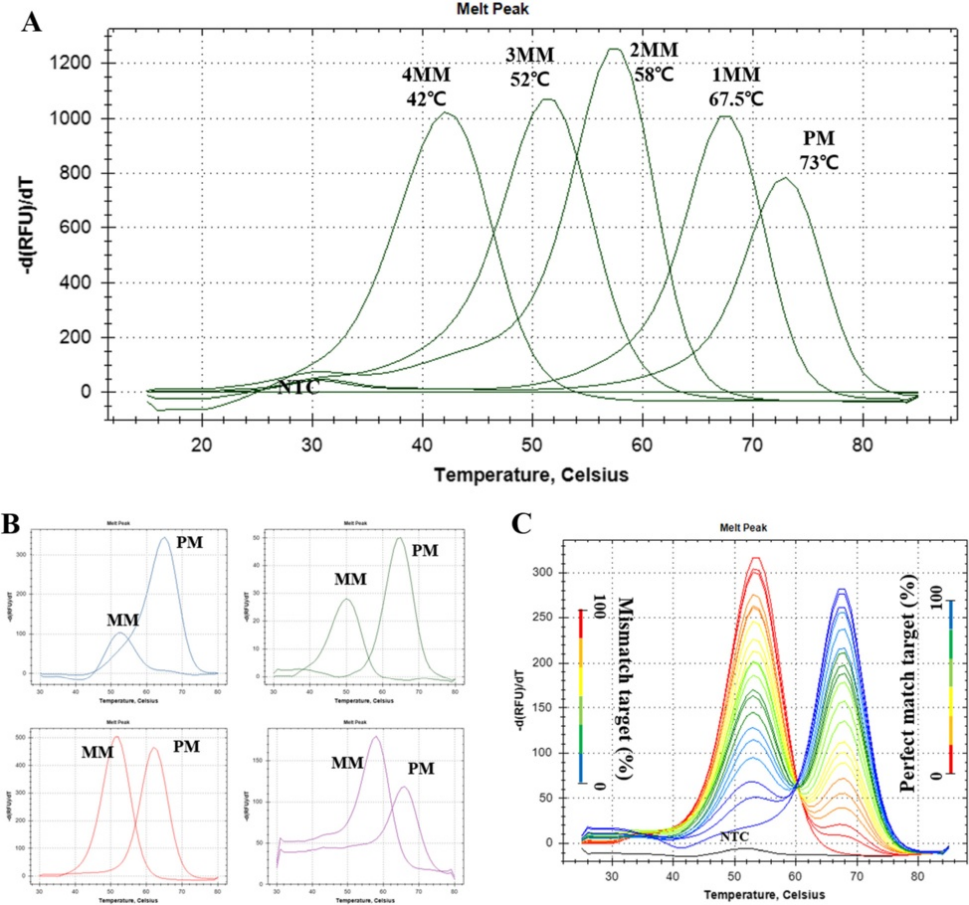
SNP detection. PNA melting point differences between perfect match and mismatch(es) were measured by melting point analysis. **a** Melting points of PNA probe and target oligonucleotide containing one to four mismatches (SNP_1probe). **b** Melting points of PNA probe containing FAM (blue line, SNP_2probe), HEX (green line, SNP_3probe), Texas-Red (red line, SNP_4probe), or Cy5 (violet line, SNP_5probe) fluorescence were analyzed after PCR cycles in closed well reactions. **c**) Melting points of synthetic DNA target with varied percentages (from 0, 5, 10, 15, 20, 25, 30, 35, 40, 45, 50, 55, 60, 65, 70, 75, 80, 85, 90, 95 to 100 %) of the mutation type of single mismatch relative to the perfect match templates using SNP_3probe. Both perfect match and single mismatch templates were artificially synthesized and start copies were roughly 2 × 10^6^ copies per reaction. PM, perfect match; 1MM, 1 nucleotide mismatch; 2MM, 2 nucleotides mismatch; 3MM, 3 nucleotides mismatch; 4MM, 4 nucleotides mismatch; NTC, non-template control

In the SNP detection study, hybridization between synthetic DNA oligomer and PNA probe was detected with melting point analysis (Fig. 2a). Melting point analysis revealed that melting point of each probe were lower than those of perfectly matched probe along with the number of mismatch with a high resolution of ΔTm (perfect match to single mismatch: 5.5 °C, single mismatch to double mismatch: 9.5 °C, double mismatch to triple mismatch: 6 °C, and triple mismatch to quadruple mismatch 10 °C). In the multi-channel SNP detection study with PCR cycle reaction, hybridization between synthetic double-stranded DNA oligomers containing four SNP loci and four PNA probes was detected with melting point analysis in one closed well (Fig. 2b). All of the fluorescence generated significant differences between perfectly matched probe signal and mismatched probes (Fig. 2b). In case of hetero-type target, mutant DNA with at least 5 % mutation could be detected with melting point analysis (Fig. 2c).

### Detection of insertion and deletion

**Fig. 3:**
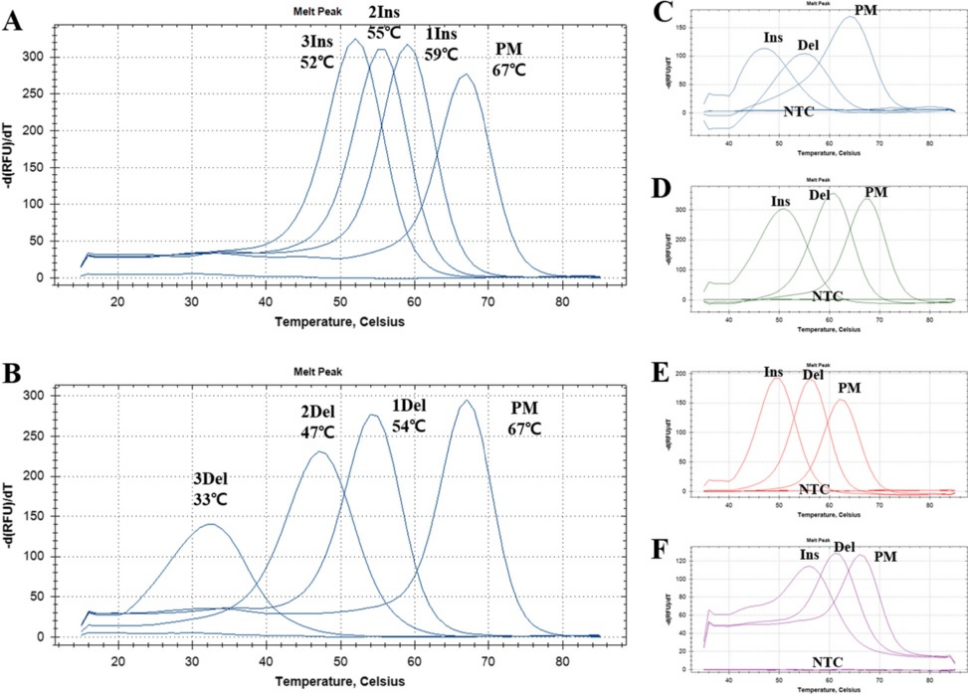
Insertion and deletion detection. PNA melting point differences between perfect match and deletion or insertion were measured by melting point analysis. **a** Melting points of PNA probe and target oligonucleotide containing one to three insertions (Indel_1probe). **b** Melting points of PNA probe and target oligonucleotide containing one to three deletions (Indel_1probe). Melting points of PNA probes containing FAM (**c**), Indel_2probe), HEX (**d**), Indel_3probe), Texas-Red (**e**), Indel_4probe), or Cy5 (**f**), Indel_5probe) fluorescence and each reaction targets containing single insertion or deletion were analyzed after PCR reaction in a closed well reaction. PM, perfect match; 1Ins, single insertion; 2Ins, double insertion; 3Ins, triple insertion; 2Del, double deletion; 3Del, triple deletion; NTC, non-template control

To detect insertion and deletion variations, hybridization between synthetic DNA oligomer and PNA probe was detected with melting point analysis. Melting point analysis revealed that melting points of each probe were lower than those of perfectly hybridized probe along with the number of insertion or deletion with a high resolution of ΔTm (perfect match 67 °C, single ins 59 °C, double ins 55 °C, triple ins 52 °C, single del 54 °C, double del 47 °C, and triple del 33 °C) (Fig. 3a and b). Analysis of insertion and deletion with PCR cycle reaction was done by hybridization between synthetic double-stranded DNA and PNA probes, which was detected with melting point analysis (Fig. 3c–f). The three synthetic double-stranded DNA targets were used as targets (PM target: four probe-binding sites with perfect match, Del: four probe-binding sites with single deletion, Ins target: four probe-binging sites with single insertion) (Additional file 2: Table S1). Deletion and insertion mutated targets revealed lowered melting points than those of perfectly matched targets in all fluorescence channels (Fig. 3c–f). In case of hetero-type target, mutant DNA with at least 5 % insertion allele could be detected with melting point analysis (Additional file 3: Figure S3).

### Multiplex mutation detection in a short target region

**Fig. 4:**
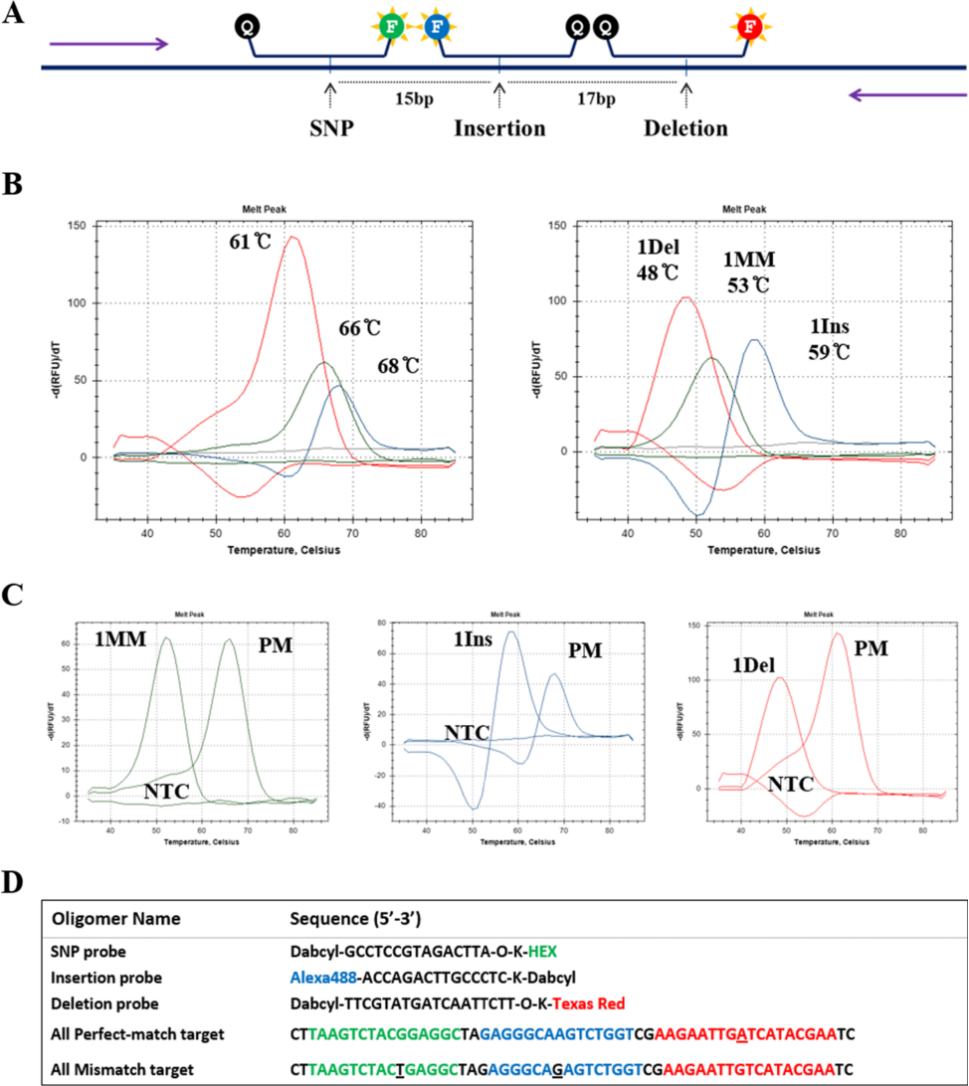
Single mutation detection in a harsh condition. PNA melting point differences between perfect match target and mutation target containing single SNP, deletion, and insertion were measured by melting point analysis. **a** Mutation points were distributed in a single amplicon with short gap with other mutation points. **b** PNA probe fluorescent signals of perfectly matched targets with three PNA probes (left panel) or melting points of PNA probe that was hybridized with single mismatch, deletion, and insertion in a single tube were represented and each signal was listed separately depending on its fluorescence (right panel). **c** Fluorescence was analyzed after PCR cycles in a closed well reaction. **d** Sequences of probes and partial targets. Underline, mismatch point; Violet arrow, primer; Red line, Texas-Red; Blue line, FAM; Green line, HEX; PM, perfect match; 1Ins, single insertion; 1Del, single deletion; NTC, non-template control

To detect narrowly distributed mutations, we used two 3’-fluorescence conjugated and one 5’-fluorescence added PNA probes to avoid fluorescence signal quenching between those probes (Fig. 4a). Three different types of mutations were artificially distributed on a single template and amplified by PCR with specific primers (Fig. 4a). All perfectly matched targets generated melting points above 60 °C (Fig. 4b) and all mismatched targets showed lower Tm values (Fig. 4c). Melting points of each probe are rearranged with its mutation (=fluorescence) (Fig. 4d).

### Quantitative analysis of PNA probe-based FMCA

**Fig. 5:**
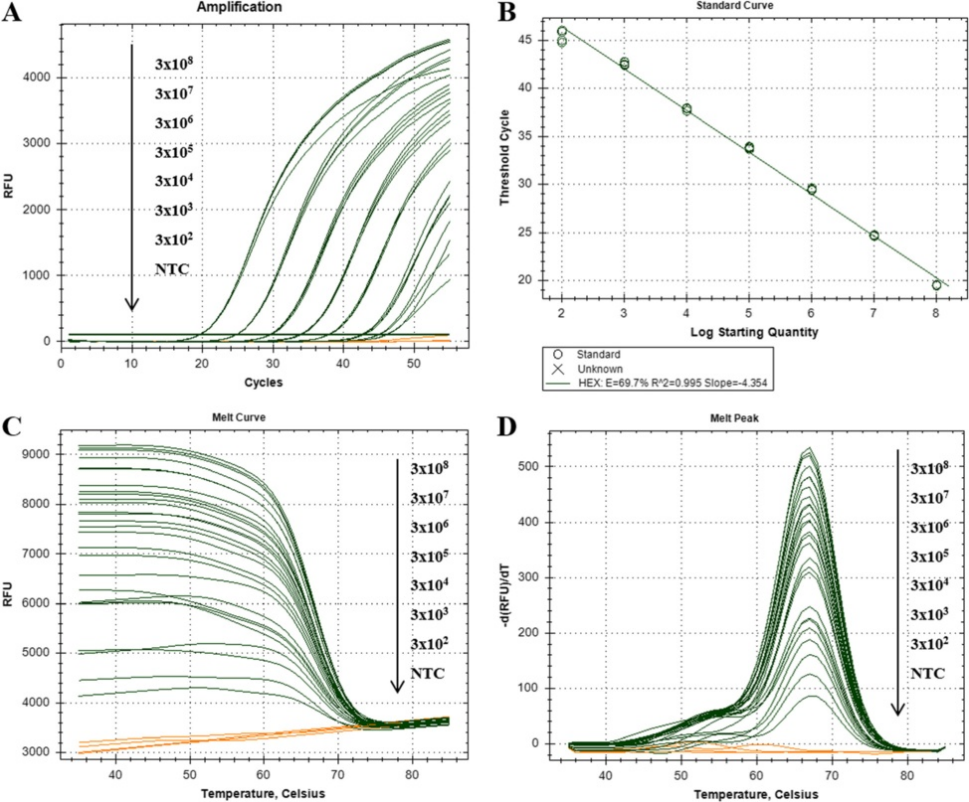
Quantitative analysis of PNA–FMCA system. Amplification points of real-time PCR reaction of FMCA conditions were measured at the primer annealing step of PCR reactions. **a** Amplification points and (**b**) standard point of each dilutant with four replicants were measured with (**c**) melting points and (**d**) melting points with four replicants in a closed well reaction. Orange lines represent negative controls

In this study, fluorescence intensity was measured at melting analysis step instead of amplification step because of its time-consuming disadvantage. To confirm the ability of measuring the PCR amplification curve and the DNA copies by PNA–fluorescence real-time PCR system, the target DNA was 10-fold diluted and amplified, and the results are shown in Fig. 5. Amplification curve (Fig. 5a) and standard curve (Fig. 5b) represent the possibility of quantitative analysis with PNA probe along with each dilutant. Analyzing quantification of initial template DNA with PNA–fluorescence real-time PCR system showed same result as performing with the traditional method measuring at amplification step (Fig. 5a–d).

### Adjustability of PNA probe hybridization temperature

**Fig. 6:**
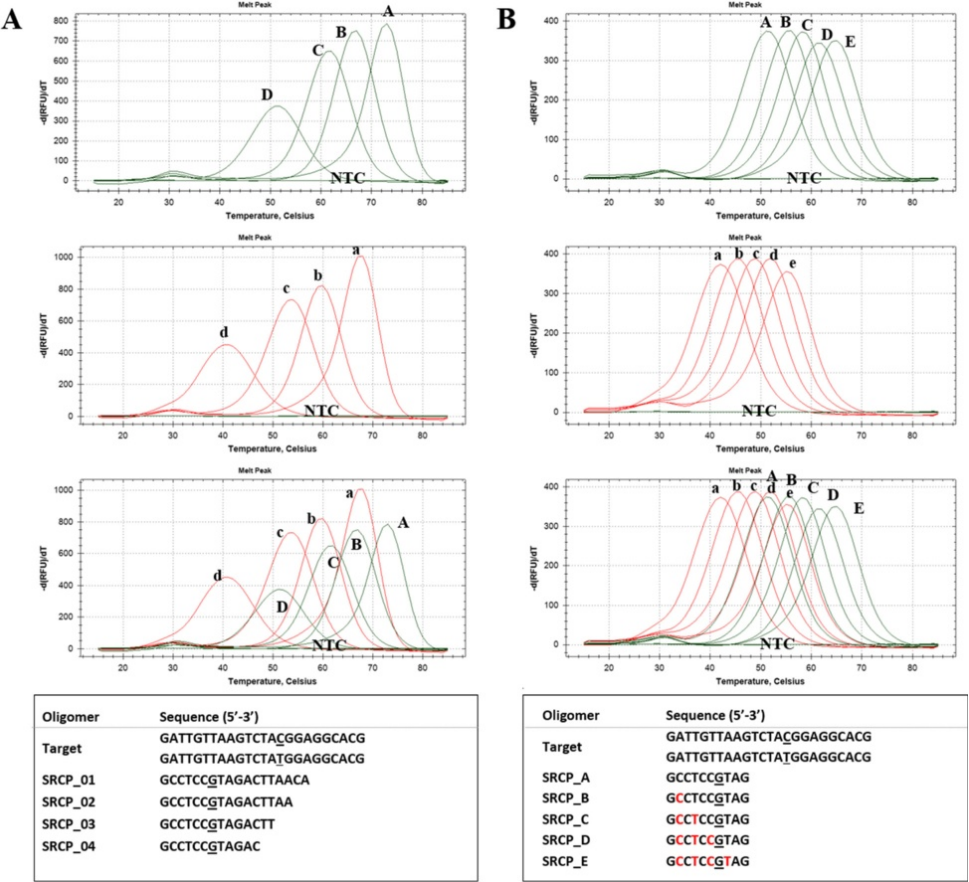
Modifiability of PNA probe Tm. **a** Melting points of PNA probes depend on probe length and (**b**) the number of gamma-modified PNA(s). Melting points of perfectly matched probes (upper panel) and single mismatch (center panel) and merged melting points (lower panel) are listed. Green lines represent perfect match and red lines represent single mismatch of each probe. Probe and target sequences are listed at the bottom of the figure. Underline, mismatch point; Red sequences, gamma-modified PNA

To detect PNA probe modifiability and adjustability of Tm value, we used two different methods to regulate Tm of PNA probe. Like DNA oligomers, Tm value radically changed depends on their length. Only 2 bp shortened PNA probe showed lowered melting point values (8 to 12 °C) and shorter length probe showed greater ΔTm between perfect and partial hybridization than that of longer (14 to 18 bp) probes (Fig. 6a). Furthermore, the Tm value of PNA probe was delicately affected by the number of gamma-modified backbone PNA oligomers showing varied ranges of Tm along with the number of gamma-modified PNA oligomers (Fig. 6b).

### Detection and discrimination between HRV and other rhabdovirus species with PNA–FMCA system *in vitro*

**Fig. 7:**
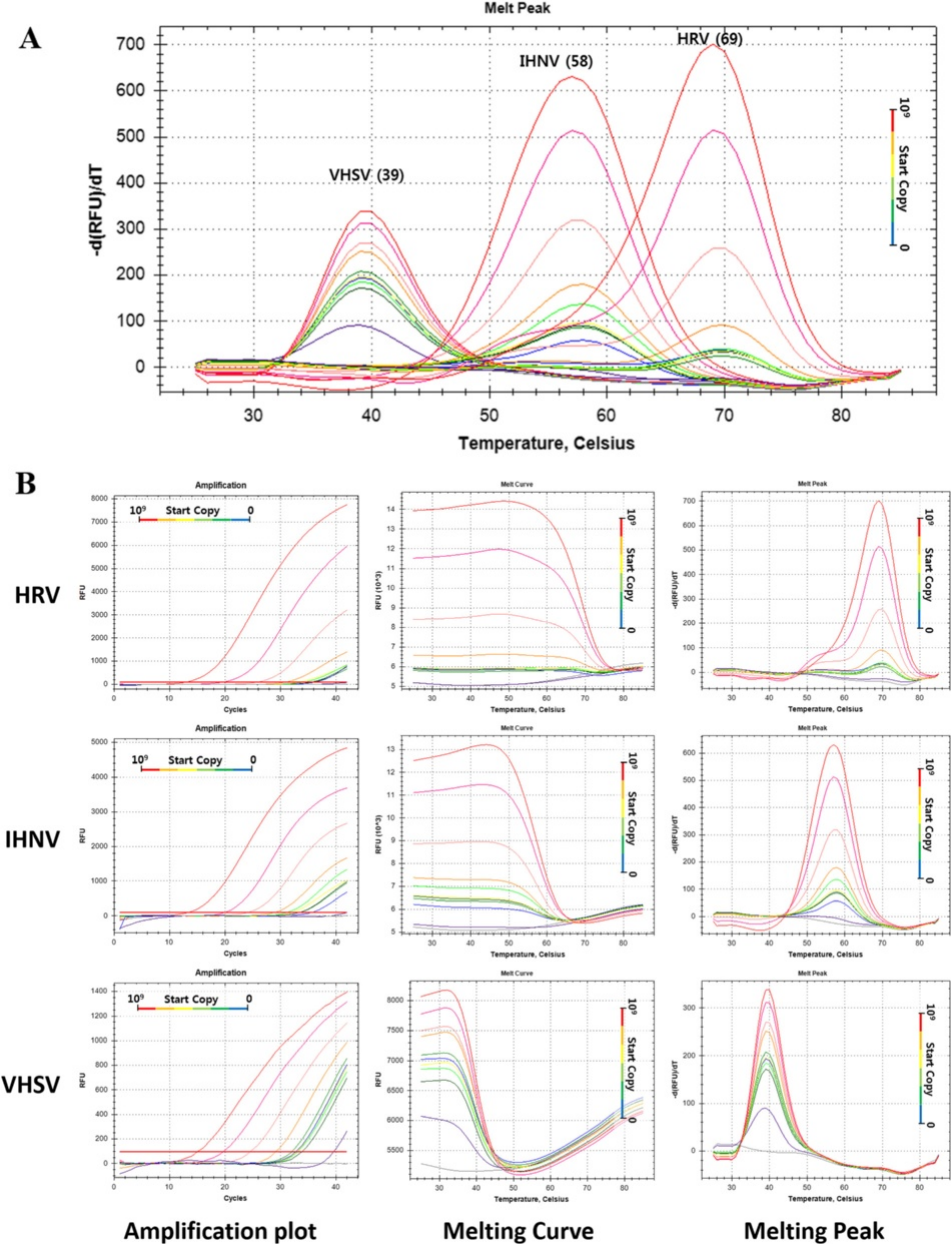
*in vitro* validation of single PNA-based FMCA for detection and discrimination between HRV and other Rhabdovirus species. The amplification plot showed quantitative detection of serially diluted targets. The melt points were converted to distinct melting points by plotting the first negative derivative of fluorescence as a function of temperature (−dF/dT). Products of different sequences were melted at different temperatures and observed as distinct points at 69 °C, 58 °C, and 39 °C)

To validate applicability of PNA-based FMCA in diagnostics, a single PNA probe targeting the specific region of glycoprotein coding sequences of three fish pathogenic viruses (HRV (CTCCAGTTGAGTGA), IHNV (CTCCAGTGGAGTGA), and VHSV (CTCCAATTGAATGA)). The PNA probe was designed to binds perfectly to HRV, with one SNP to IHNV and with two SNP to VHSV. The real-time PCR result showed that Tm of PNA probe depends on each species with HRV (69 °C), IHNV (58 °C), and VHSV (39 °C) (Fig. 7a). Furthermore, this probe melting point analysis revealed the possibility of quantification detection with 10-fold diluted synthetic DNA targets (10^9^ to 1 start copies) (Fig. 7b).

## Discussion

There are many ways to genotype nucleotide differences or changes in genome [11]. Currently available techniques that require a separation step include restriction fragment length polymorphism analysis, single-nucleotide extension, oligonucleotide ligation, and direct sequencing. Additional methods, including pyrosequencing [12] and mass spectroscopy [13], are technically complex but can be automated for high-throughput analysis. Furthermore, real-time PCR-based assays that use high resolution melting (HRM) and probe-based systems, FRET probes, molecular beacons, or TaqMan probes have been adapted for continuous mutation detection of amplified products in a closed system. However, their capacity to discriminate DNA variants in the single nucleotide indel as well as SNP variant with single probe has not been examined until now. Furthermore, traditional probe systems that have been used in SNP detection use two or more fluorescence channels for one mutation loci. Thus even a wide range of mutation detection methodologies exist, DNA sequencing technology has been considered as the gold standard. However, several drawbacks have been reported in DNA sequencing, including its interpretation errors [14] and difficulty of high-throughput data generation, which makes DNA sequencing difficult to use. To solve these problems, we have adopted the full base PNA hybridization probes [9] for characterization of genetic variation assays.

The melting temperature (Tm) of nucleic acid oligomer is a physical parameter of nucleic acid hybrid. Under constant reaction conditions of heating rate, salt concentration, and probe-target concentrations, Tm is highly reproducible. Recently, HRM [15] and DNA, LNA probe melting analysis have been introduced and used for genotyping studies [16]. However, multiplexing obtained by HRM alone will be limited and probe-based melting technologies face a difficulty of probe design, and low resolution (temperature difference) of variants remains to be limited by using existing probe chemistries (DNA, LNA. etc.).

To solve these problems, we adopted full base peptide nucleic acid (PNA) oligomer to probe-based melting point analysis. PNAs are artificially synthesized DNA analogs with an uncharged peptide backbone. PNAs have more favorable hybridization properties because of their uncharged nature and their peptide bond-linked backbone [8]. Because of these favorable characteristics, PNA probes are able to be designed shorter (9–13 bp) than DNA probes (20 bp or more) with the same Tm. These characteristics make PNA probe more acceptable to use in genetic variation detection studies because PNA probe makes a large difference in ΔTm between perfect match and partial mismatch, including even insertion and deletion. In this study, PNA probes were used dual-labeled probe-based real-time PCR melting point anlysis to analyze genetic mutations include SNP, insertion, and deletion for genetic difference detection. The developments in self-quenching PNA real-time PCR probe have played a crucial role in the emergence of PNA probe-based melting analysis.

In here, we have shown that PNA probe-based melting system is a suitable method for nucleic acid change detection, as it was able to sensitively discriminate insertion(s), deletion(s), and SNP(s) along with the number of variations (Figs. 2 and 3). In case of narrowly distributed mutations (three mutations within 32 bp) (Fig. 4), HRM or DNA probe¬-based melting point assay may show erroneous results, because one amplicon or DNA probe contains multiple mutations that counteract each other, which causes differences in the Tm [17]. In this study, the problem was completely resolved using dual-labeled PNA probes with two 5’-end quencher and 3’-end fluorescence conjugated PNA probe and one reversely conjugated PNA probe (Fig. 4a).

In addition to the genotyping studies, we have characterized PNA probe and asymmetric PCR conditions to verify their applicability. Genotyping analysis was measured without amplification measuring steps because of its time-consuming disadvantage. Therefore, to confirm the ability to measure the PCR amplification curve and DNA copies by dual-labeled PNA probe system, the target DNA was 10-fold diluted and amplified. Amplification curve (Fig. 5a) and standard curve (Fig. 5b) represent the possibility of quantitative analysis with dual-labeled PNA probe corresponding to its dilution ratios. This applicability was further confirmed by virus g-protein gene analogues detection study (Fig. 7). Furthermore, to detect amplification curve in the PNA-based probe system for quantitative analysis, the detection temperature depends on the primer annealing temperature. If probe-binding temperature is lower than that of the primer, the detection step will be added to detect fluorescence signal of lower Tm probe [18].

Moreover, shorter probe length with higher Tm was highly recommended due to the fact that shorter probe length makes ΔTm between perfect match and mismatch greater. Therefore, modifiability of probe Tm value plays a pivotal role in probe-based melting point analysis. We propose that there are two different ways to regulate Tm of a PNA probe. First of all, Like DNA oligomers, the Tm value of PNA probe radically depends on their length (Fig. 6a). Secondly, PNA oligomer is easily modifiable because of its peptide backbone [8]. Gamma-modified PNA oligomer offers structural formation and ionic positive charges, which make PNA probe-binding efficiency higher [8]. In this study, the Tm value of PNA probe was delicately affected by the number of gamma (Ala)-modified PNA oligomers of probe sequences (Fig. 6b). The PNA probes have varied ranges of Tm along with the number of gamma (Ala)-modified PNA oligomers (approximately 3–15 °C).

## Conclusions

Here we show that peptide nucleic acid (PNA) is apt material for real-time PCR fluorescence probe for melting point analysis because it makes a large difference in the melting temperature (ΔTm) between full specific hybridization and that of single mismatch, including insertion and deletion. Furthermore, it is possible that PNA fluorescence probe effectively classify the co-existed SNP, insertion and deletion within short amplicon (32 bp) without any interference, which completely resolve the problem of high resolution melting (HRM) genotyping method. Moreover, applicability of PNA fluorescence probe is confirmed by the gamma modification of PNA that can simply substitute minor groove binder (MGB) of DNA probe with specific hybridization. Taken together, these characteristics demonstrated that dual-labeled and gamma-modified PNA probe greatly simplifies the probe design and dramatically increases allele-discriminating ability.

## Methods

### PNA oligomer and DNA target

All PNA probes (FAM-, HEX-, Texas Red-, or Cy5-labeled with quencher) were purified using high-performance liquid chromatography (Panagene, Korea), and the target oligonucleotides were synthesized and purified using polyacrylamide gel electrophoresis (Neoprobe, Korea). The purity of all the probes was confirmed by mass spectrometry. Unwanted secondary structure in the probe was avoided for better hybridization with its target. The mutation points were basically located in the center of the probe so as to obtain a Tm shift over 5 °C. For detection of deletion and insertion, end position deletion/insertion and center position deletion/insertion were used to optimize Tm shift. Single-stranded DNA oligomers were used as target DNA for probe validation, and each target DNA contained mismatch(es), deletion(s), or insertion(s). For mutation detection using PCR cyclic steps, synthetic double-stranded DNA containing two primers and probe-binding site(s) was used as the target. Four synthetic double-stranded DNA targets were randomly designed with 80 bp of DNA, containing one probe and two primer-binding sites, to analyze the ability to detect SNP. To analyze the differences in Tm between perfect match and deletion or insertion, three double-stranded DNA targets containing four perfectly matched probe-binding sites and four deletion or insertion sites in a single amplicon were artificially synthesized because of the cost efficiency in synthesizing double-stranded DNA. To analyze detection ability in a harsh condition, target DNA containing one SNP, deletion, and insertion in 118 bp DNA with a gap of 15 and 17 bp between each mutation point was synthesized. To avoid quenching of fluorescence signal within other probes, fluorescence and dabcyl (quencher) positions of Alexa488-probe were switched. The PNA oligomer, DNA target, and primer sequences are listed in Additional file 2: Table S1.

### Direct analysis of PNA probe Tm value using fluorescence melting point analysis (FMCA) with synthetic oligonucleotides

Hybridization between probes and synthetic oligonucleotide targets was performed in a CFX96™ Real-Time System (BIO-RAD, USA). To analyze the Tm of PNA probe, synthetic single-stranded DNA oligomers were directly used as the target DNA. A thermal cyclic reaction was performed using the following conditions: the 20 μL reactions contained 1X SSB Real-Time FMCA™ buffer (SeaSun Biomaterials, Korea), 0.5 μM PNA probe, and 0.5 μL of DNA template (1 × 10^5^ copies of synthetic DNA). Melting point analysis began with a denaturation step of 3 min at 95 °C; a stepwise hybridization and followed by a stepwise temperature increase up to 85 °C at 0.5 °C/step with a 5 s interval between each step (Additional file 4: Figure S1). The target oligomers and probes used are listed in Additional file 2: Table S1.

### PNA probe-based FMCA for detection of genetic variation with PCR amplification

PCR and thermal cyclic reaction and hybridization between probes and synthetic oligonucleotide targets were performed in a CFX96™ Real-Time System (BIO-RAD, USA). In all the PCR amplification conditions, asymmetric PCR was used to generate single-stranded DNA target. An asymmetric PCR was carried out using the following conditions: the 20 μL reactions contained 1X SSB Real-Time FMCA™ buffer (SeaSun Biomaterials, Korea), 2.5 mM MgCl_2_, 200 μM dNTPs, 1.0 U Taq polymerase, 0.05 μM forward primer and 0.5 μM reverse primer, 0.5 μM PNA probe, and 0.5 μL of DNA template (1 × 10^5^ copies of synthetic DNA). Real-time PCR and FMCA protocols started with a denaturation step of 7 min at 95 °C, followed by 32 ~ 50 cycles of 95 °C for 10 s, 55 °C for 15 s, and 74 °C for 30 s. Melting point analysis began with a denaturation step of 3 min at 95 °C, hybridization step, and followed by a stepwise temperature increase from 25, 30, or 35 °C to 85 °C at 1 °C/step with a 5 s interval between each step (Additional file 4: Figure S1). The forward and reverse primers and probes used are listed in Additional file 2: Table S1.

### Hetero-type variation detection with PNA-based FMCA

To analyze hetero-type variation detection, DNA template consists of mutation type SNP was mixed with DNA does not consist of the SNP, with varied percentages (from 0, 5, 10, 15, 20, 25, 30, 35, 40, 45, 50, 55, 60, 65, 70, 75, 80, 85, 90, 95 to 100 %) and used as a template. Mutant-type target DNA was serially diluted in wild-type target DNA. Both perfect match and mutation templates were artificially synthesized and start copies were roughly 2 × 10^5^ copies per reaction. The PNA-based FMCA was performed according to the conditions mentioned above.

### Multiple mutation detection in a short target region

Multiple mutation detection was performed using the following conditions: the 20 μL reactions contained 1X SSB Real-Time FMCA™ buffer, 2.5 mM MgCl_2_, 200 μM dNTPs, 1.0 U Taq polymerase, 0.05 μM forward primer and 0.5 μM reverse primer, 0.5 μM probe per each fluorescence (Alexa488, HEX, Texas-red), and 0.5 μL of DNA template (1 × 10^5^ copies of synthetic DNA). The same real-time PCR and FMCA protocols mentioned above were used.

### Quantitative analysis of PNA-based FMCA

Serially diluted DNA template (1 × 10^8^ to 1 × 10^2^ copies of synthetic DNA) was used for quantitative analysis of PNA-based FMCA. Real-time PCR and FMCA protocols were performed as per the conditions mentioned above.

### Detection and discrimination between HRV and other rhabdovirus species with PNA–FMCA system *in vitro*

Self-quenching PNA probe was designed (Dabcyl-TCACTCAACTGGAG-Texas-Red) using specific region of the glycoprotein of HRV (CTCCAGTTGAGTGA), IHNV (CTCCAGTGGAGTGA), and VHSV (CTCCAATTGAATGA) sequences that was discriminated by perfect match (HRV), single mismatch (IHNV), or two mismatches (VHSV). Synthetic DNA (276 bp) targets were amplified using PCR and hybridized with the chosen PNA probe containing an SNP site(s) mid-sequence. The PNA-based FMCA was performed as per the conditions mentioned above. The sensitivity test of this procedure was conducted using 10-fold-diluted synthetic DNA targets (10^9^ to 1 start copies).

## Additional files

Additional file 1: Figure S2. Insertion detection. PNA melting peak differences between perfect match and insertion were measured by melting curve and peak analysis. A. Melting curves (left) and melting peaks (right) of PNA probe and target oligonucleotide that contains one insertion to three insertions with detection type I (sequence shift). B. Melting curves (left) and melting peaks (right) of PNA probe and target oligonucleotide that contains one insertion to three insertions with detection type II (structural difference). PM, perfect match; Ins, single insertion; NTC, negative control. (PNG 290 kb) Additional file 2: Table S1. DNA and PNA oligomer Sequences. (DOCX 21 kb) Additional file 3: Figure S3. Melting points of hetero-type insertion. PNA melting point differences between perfect match and single insertion were measured by melting point analysis. Melting points of synthetic DNA target with varied percentages (from 0, 5, 10, 15, 20, 25, 30, 35, 40, 45, 50, 55, 60, 65, 70, 75, 80, 85, 90, 95 to 100%) of the mutation type of single insertion relative to the perfect match templates using Indel_3probe. Both perfect match and single mismatch templates were artificially synthesized and start copies were roughly 2x10^6^ copies per reaction. PM, perfect match; 1MM, 1 nucleotide mismatch; 2MM, 2 nucleotides mismatch; 3MM, 3 nucleotides mismatch; 4MM, 4 nucleotides mismatch; NTC, non-template control. (PNG 270 kb) Additional file 4: Figure S1. Real-time PCR run information. Melting point analysis and/or PCR amplification signals were conducted by specific real-time PCR condition. **A**) Direct analysis of PNA probe Tm value using fluorescence melting point analysis (FMCA) with synthetic oligonucleotides. **B**) Hetero-Type SNP detection with PNA-Based FMCA. **C**) Multiple mutation detection in a short target region. **D**) Quantitative analysis of PNA-based FMCA. (PNG 414 kb)
