# Supplementary figures and images for: Detection of genetic variation using dual-labeled peptide nucleic acid (PNA) probe-based melting point analysis
Source: Biol Proced Online. 2015 Nov 4;17:14. doi: 10.1186/s12575-015-0027-5 (PMC4632671; doi:10.1186/s12575-015-0027-5)

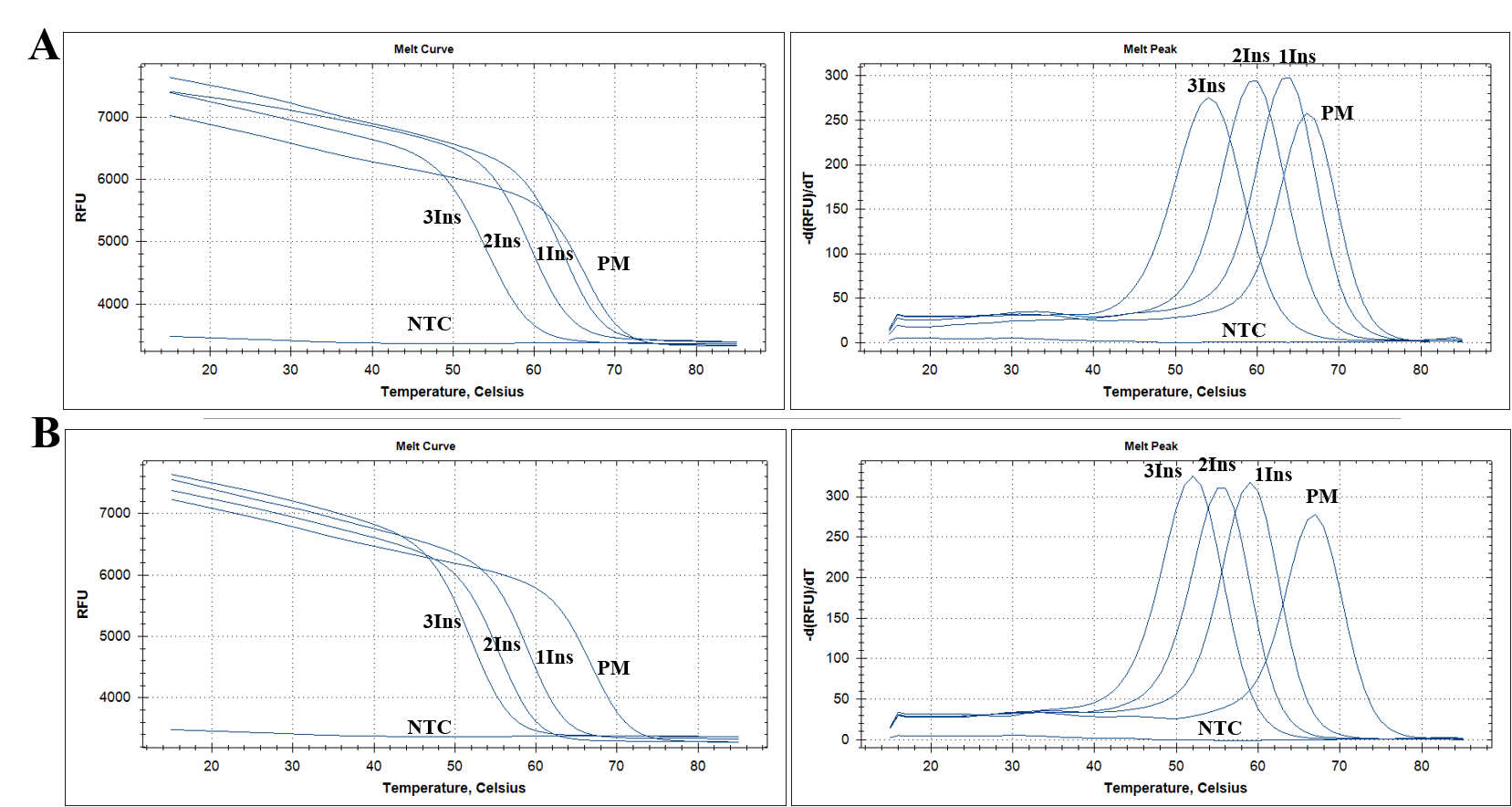

Supplement: Additional file 1: Figure S2. — Insertion detection. PNA melting peak differences between perfect match and insertion were measured by melting curve and peak analysis. A. Melting curves (left) and melting peaks (right) of PNA probe and target oligonucleotide that contains one insertion to three insertions with detection type I (sequence shift). B. Melting curves (left) and melting peaks (right) of PNA probe and target oligonucleotide that contains one insertion to three insertions with detection type II (structural difference). PM, perfect match; Ins, single insertion; NTC, negative control. (PNG 290 kb) [file 12575_2015_27_MOESM1_ESM.png]

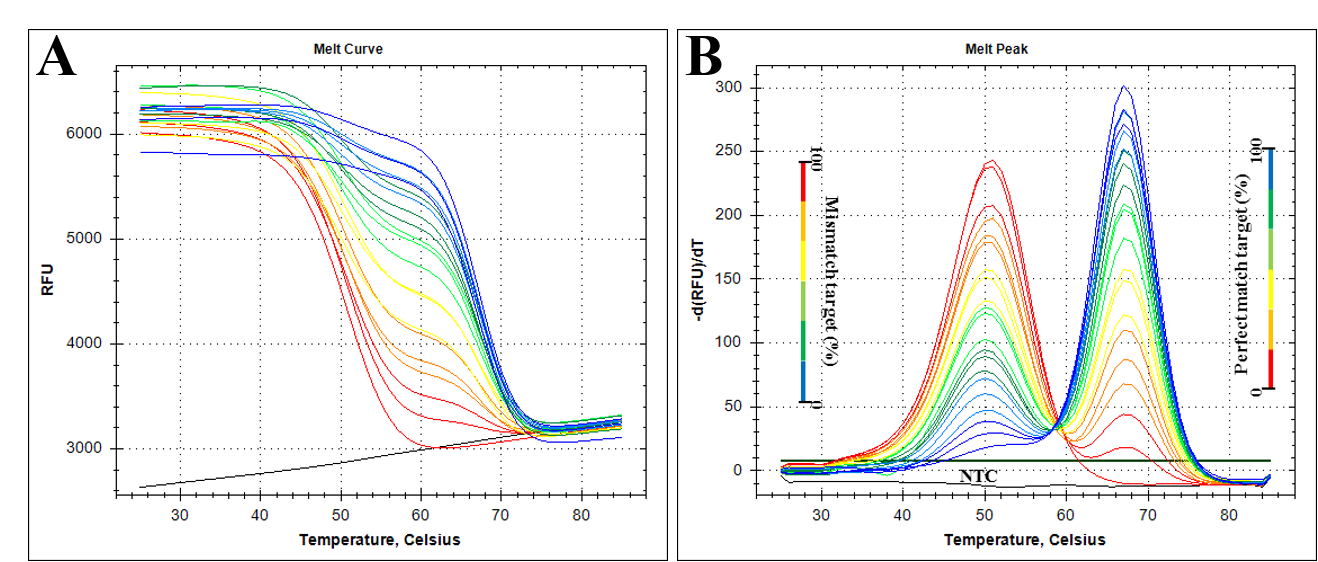

Supplement: Additional file 3: Figure S3. — Melting points of hetero-type insertion. PNA melting point differences between perfect match and single insertion were measured by melting point analysis. Melting points of synthetic DNA target with varied percentages (from 0, 5, 10, 15, 20, 25, 30, 35, 40, 45, 50, 55, 60, 65, 70, 75, 80, 85, 90, 95 to 100%) of the mutation type of single insertion relative to the perfect match templates using Indel_3probe. Both perfect match and single mismatch templates were artificially synthesized and start copies were roughly 2x10^6^ copies per reaction. PM, perfect match; 1MM, 1 nucleotide mismatch; 2MM, 2 nucleotides mismatch; 3MM, 3 nucleotides mismatch; 4MM, 4 nucleotides mismatch; NTC, non-template control. (PNG 270 kb) [file 12575_2015_27_MOESM3_ESM.png]

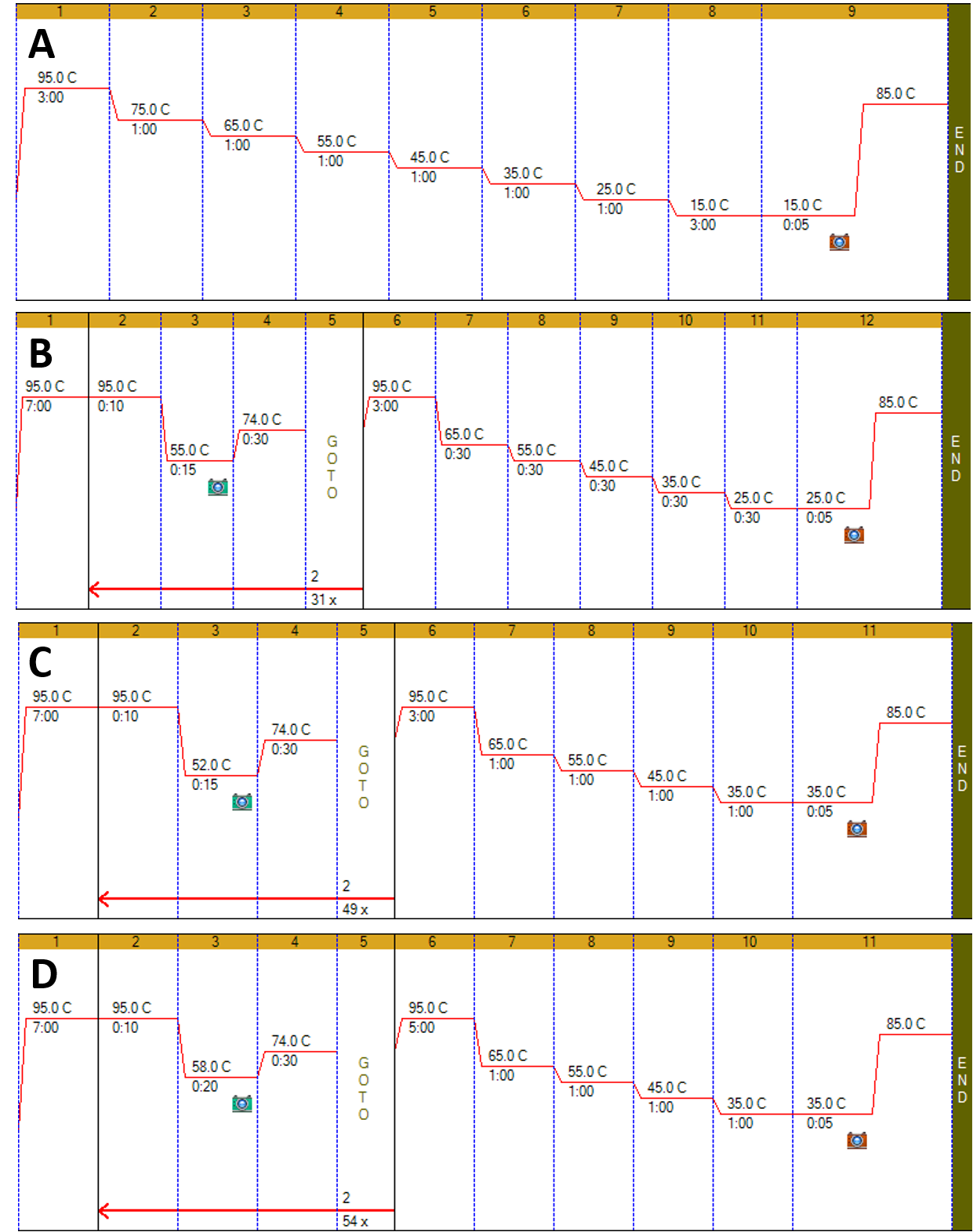

Supplement: Additional file 4: Figure S1. — Real-time PCR run information. Melting point analysis and/or PCR amplification signals were conducted by specific real-time PCR condition. A) Direct analysis of PNA probe Tm value using fluorescence melting point analysis (FMCA) with synthetic oligonucleotides. B) Hetero-Type SNP detection with PNA-Based FMCA. C) Multiple mutation detection in a short target region. D) Quantitative analysis of PNA-based FMCA. (PNG 414 kb) [file 12575_2015_27_MOESM4_ESM.png]
