# Supplementary material for: Detection of genetic variation using dual-labeled peptide nucleic acid (PNA) probe-based melting point analysis
Source: Biol Proced Online. 2015 Nov 4;17:14. doi: 10.1186/s12575-015-0027-5 (PMC4632671; doi:10.1186/s12575-015-0027-5)
Supplement: Additional file 2: Table S1. — DNA and PNA oligomer Sequences. (DOCX 21 kb) [file 12575_2015_27_MOESM2_ESM.docx]

| **Table S1. DNA and PNA oligomer Sequences** | | | | | | |
| --- | --- | --- | --- | --- | --- | --- |
| **Applications** | | | | **Target DNA (5’-3’)** | **PNA Probe (Name: Sequence 5’-3’)** | **Primer F/R(5’-3’)** |
| SNP | Oligo | | PM | GATTGTTAAGTCTACGGAGGCACG | SNP_1: Dabcyl-GCCTCCGTAGACTTAACA-O-K(HEX) |  |
|  |  |  | 1MM | GATTGTTAAGTCTATGGAGGCACG |  |  |
|  |  |  | 2MM | GATTGTTAAGCCTATGGAGGCACG |  |  |
|  |  |  | 3MM | GATTGTTTAGCCTATGGAGGCACG |  |  |
|  |  |  | 4MM | GATTGTTTAGCCTATGGTGGCACG |  |  |
|  | DS-DNA | | PM | AATACGACTCACTATAGGGGGCCGGAACAGGTTGAACTGTGTACCCCCCCTTGGCAGGAAATCTATAGTGTCACCTAAAT | SNP_2: Dabcyl-GTTGAACTGTGTACCC-O-K(FAM) | AATACGACTCACTATAG/  ATTTAGGTGACACTATAG |
|  |  |  | MM | AATACGACTCACTATAGGGGGCCGGAACAGGTTGAACTGTTTACCCCCCCTTGGCAGGAAATCTATAGTGTCACCTAAAT |  |  |
|  |  |  | PM | GTTTTCCCAGTCACGACTATAGCCCACGCTGGGGCCTCCGTAGACTTAACAATTTTCTCCCTTGTCATAGCTGTTTCCTG | SNP_3: Dabcyl-GCCTCCGTAGACTTA-O-K(HEX) | GTTTTCCCAGTCACGAC/  CAGGAAACAGCTATGAC |
|  |  |  | MM | GTTTTCCCAGTCACGACTATAGCCCACGCTGGGGCCTCAGTAGACTTAACAATTTTCTCCCTTGTCATAGCTGTTTCCTG |  |  |
|  |  |  | PM | GTAAAACGACGGCCAGTCCAAACACCTTTATTCGTATGATCAATTCTTGTTACAACTGCCTGTGTGAAATTGTTATCCGC | SNP_4: Dabcyl-TTCGTATGATCAATTCTT-O-K(Texas Red) | GTAAAACGACGGCCAGT/  GCGGATAACAATTTCACACAGG |
|  |  |  | MM | GTAAAACGACGGCCAGTCCAAACACCTTTATTCGTGTGATCAATTCTTGTTACAACTGCCTGTGTGAAATTGTTATCCGC |  |  |
|  |  |  | PM | GGCAAGCCACGTTTGGTGGTTACAACTGTCTTGCTTCTTATGGCCCTCCCAGTCCTAGCACCTCTGACACATGCAGCTCC | SNP_5: Dabcyl-CTTCTTATGGCCCTCCC-O-K(Cy5) | GGCAAGCCACGTTTGGTG/  GGAGCTGCATGTGTCAGAGG |
|  |  |  | MM | GGCAAGCCACGTTTGGTGGTTACAACTGTCTTGCTTCTTATAGCCCTCCCAGTCCTAGCACCTCTGACACATGCAGCTCC |  |  |
| Deletion/ Insertion | Oligo | Del | PM | ATGAGGGTACACAGTTCAACCAG | Indel_1: Dabcyl-GTTGAACTGTGTACCC-O-K(FAM) |  |
|  |  |  | 1Del | TATGAGGTACACAGTTCAACCAG |  |  |
|  |  |  | 2Del | ATATGAGTACACAGTTCAACCAG |  |  |
|  |  |  | 3Del | TATATGATACACAGTTCAACCAG |  |  |
|  |  |  | PM | TATAGGGTACACAGTTCAACCAG |  |  |
|  |  |  | 1Del | CTATAGGGTACCAGTTCAACCAG |  |  |
|  |  |  | 2Del | CTATAGGGTACAGTTCAACCAGC |  |  |
|  |  |  | 3Del | CTATAGGGTAAGTTCAACCAGCA |  |  |
|  |  | Ins | PM | CTATATGAGGGTACACAGTTCAACCG |  |  |
|  |  |  | 1Ins | TATATGAGAGGTACACAGTTCAACCG |  |  |
|  |  |  | 2Ins | ATATGAGGTAGTACACAGTTCAACCG |  |  |
|  |  |  | 3Ins | TATGAGGGATATACACAGTTCAACCG |  |  |
|  |  |  | PM | CTATAGGGTACACAGTTCAACCAG |  |  |
|  |  |  | 1Ins | TATAGGGTACAGCAGTTCAACCAG |  |  |
|  |  |  | 2Ins | ATAGGGTACATGCAGTTCAACCAG |  |  |
|  |  |  | 3Ins | TAGGGTACAATGCAGTTCAACCAG |  |  |
|  | DS-DNA | | PM | GGCTCAGCCATCTTACCTGTGGCACAGGTTGAACTGTGTACCCCCCCTTCGTTGGGGCCTCCGTAGACTTAACAACTTTATTCGTATGATCAATTCTTGTGTCTTGCTTCTTATGGCCCTCCCAGTTTCTGATTCTTCGGACACCCGGC | Indel_2: Dabcyl-GTTGAACTGTGTACCC-O-K(FAM)  Indel_3: Dabcyl-GCCTCCGTAGACTTA-O-K(HEX)  Indel_4: Dabcyl-TTCGTATGATCAATTCTT-O-K(Texas Red)  Indel_5: Dabcyl-CTTCTTATGGCCCTCCC-O-K(Cy5) | TCAGCCATCTTACCTGTGGC/  GGGTGTCCGAAGAATCAGAA |
|  |  |  | Del | GGCTCAGCCATCTTACCTGTGGCACAGGTTGAACTGGTACCCCCCCTTCGTTGGGGCCTCGTAGACTTAACAACTTTATTCGTAGATCAATTCTTGTGTCTTGCTTCTTTGGCCCTCCCAGTTTCTGATTCTTCGGACACCCGGC |  |  |
|  |  |  | Ins | GGCTCAGCCATCTTACCTGTGGCACAGGTTGAACTGCTGTACCCCCCCTTCGTTGGGGCCTCGCGTAGACTTAACAACTTTATTCGTATTGATCAATTCTTGTGTCTTGCTTCTTAATGGCCCTCCCAGTTTCTGATTCTTCGGACACCCGGC |  |  |
| Inter-multiple | Oligo | | PM | CTTAAGTCTACGGAGGCTAGAGGGCAAGTCTGGTCGAAGAATTGATCATACGAATC | Inter_1: Alexa488-ACCAGACTTGCCCTC-K(Dabcyl)  Inter_2: Dabcyl-GCCTCCGTAGACTTA-O-K(HEX)  Inter_3: Dabcyl-TTCGTATGATCAATTCTT-O-K(Texas Red) |  |
|  |  | | MM | CTTAAGTCTACTGAGGCTAGAGGGCAGAGTCTGGTCGAAGAATTGTCATACGAATC |  |  |
|  | DS-DNA | | PM | GTCCGTAGGTGAACCTGCGGAATGGTCGGACTGCCTCCGTAGACTTATAACCAGACTTGCCCTCCGTTCGTATGATCAATTCTTTCTCGGGCAGAACGCATATCAATAAGCGGAGGAG |  | TCAGCCATCTTACCTGTGGC/  GGGTGTCCGAAGAATCAGAA |
|  |  | | MM | GTCCGTAGGTGAACCTGCGGAATGGTCGGACTGCCTCAGTAGACTTATAACCAGACTCTGCCCTCCGTTCGTATGACAATTCTTTCTCGGGCAGAACGCATATCAATAAGCGGAGGAG |  |  |
| PNA probe Characterization | Oligo | | PM | GATTGTTAAGTCTACGGAGGCACG | Cha_1: Dabcyl-GCCTCCGTAGACTTAACA-O-K(HEX) |  |
|  |  |  | MM | GATTGTTAAGTCTATGGAGGCACG | Cha_2: Dabcyl-GCCTCCGTAGACTTAA-O-K(HEX) |  |
|  |  |  |  |  | Cha_3: Dabcyl-GCCTCCGTAGACTT-O-K(HEX) |  |
|  |  |  |  |  | Cha_4: Dabcyl-GCCTCCGTAGAC-O-K(HEX) |  |
|  |  |  |  |  | Cha_5: Dabcyl-GCCTCCGTAG-O-K(HEX) |  |
|  |  |  |  |  | Cha_6: Dabcyl-GC*CTCCGTAG-O-K(HEX) |  |
|  |  |  |  |  | Cha_7: Dabcyl-GC*CT*CCGTAG-O-K(HEX) |  |
|  |  |  |  |  | Cha_8: Dabcyl-GC*CT*CC*GTAG-O-K(HEX) |  |
|  |  |  |  |  | Cha_9: Dabcyl-GC*CT*CC*GT*AG-O-K(HEX) |  |
| Virus discrimination | VHSV | | 2MM | AGTGGAATGCTCGTTCAAAGAAACCTTGTGGAAGTCCCCCATCTGAGCATTGTGTTTGTCTCCAACACATCTGATCTTTCCACCAATTACATCCACACCAACCTAATCCCCTCGGATTGGTCATTCCACTGGAGTCTTTGGCCATCATTATCGGGGATGGGGGTTGTGGGAGGGGCCTTCCTTCTACTGGTACTCTGCTGTTGCTGCAAGGCGTCCCCTCCCATTCCAAACTACGGGATTCCGATGCAGCAGTTCTCCAGAAGTCAGACGGTCTGA | Virus_1: Dabcyl-TCACTCAACTGGAG-O-K(Texas Red) | GTTCGTCTCCAACACATCTGATC/TGGAGAACTGCTGCATCGGA |
|  | IHNV | | 1MM | TGAGCATGATGGAACCGATGAGCATCAAATCCGTACCCCATCCGAGCATCCTGGACTTCTACAATGAGACAGACGTATCTGGGATCTCCATCAGGAAATTGGACTCGTTCGACCTTCAATCACTCCACTGGAGTTTCTGGCCCACAATCTCCACCCTGGGTGGGGTTCCCCTGGTTCTCCTCCTTGCTGTTGCCGCGTGCTGCTGCTGGTCAGGGAGACCTCCCACTCCTTCTGCGCCGCAGAGCATCCCCATGTATCACCTGGCAAACCGGTCCT |  | ATGGAACCGATGAGCATCAAATC/GTTTGCCAGGTGATACATGGGG |
|  | HRV | | PM | AAGGTGAGCATGATGGACAAGATGGACATTCGCCCCGTTCCGCATCCTAGTGTCCAGATACTCTACAACGACACAGACACCGCAGACATCACAATCAGGAAGATAGACTCGTTTGATCTGCAATCACTCAACTGGAGCTTCTGGCCATCATTGTCAGCACTGGGAGGGGTTCCAATACTCCTCGCCCTCGTATTCTTTCTGTACTGCTGCATGAACAGAAGACCCTCCATGCCTGCAGCACCCCAAGAGATCCCCATGTACCACCTCGCCAGTCGA |  | ATGGACAAGATGGACATTCGCC/ AGGTGGTACATGGGGATCTCTT |

Oligo, single stranded DNA; DS-DNA, double stranded DNA; PM, perfect match; 1MM, 1 nucleotide mismatch; 2MM, 2 nucleotides mismatch; 3MM, 3 nucleotides mismatch; 4MM, 4 nucleotides mismatch; 1Ins, single insertion; 2Ins, double insertion; 3Ins, triple insertion; 1Del, single deletion; 2Del, double deletion; 3Del, triple deletion; *, gamma modified PNA.
